# Choledochal Cyst in Children, presentation and outcome - 10 years’ experience from a tertiary care center in Pakistan

**DOI:** 10.12669/pjms.39.2.6196

**Published:** 2023

**Authors:** Muhammad Arslan Farooq, Sabeen Abid Khan, Munir Iqbal Malik

**Affiliations:** 1 Dr. Muhammad Arslan Farooq, MBBS, FCPS (Pediatrics). Senior Registrar, Pediatrics, Shifa Tameer-e-Millat University (Shifa College of Medicine)/ Shifa International Hospital, Islamabad, Pakistan; 2 Dr. Sabeen Abid Khan, MBBS, FCPS (Pediatrics). Associate Professor, Pediatrics, Shifa Tameer-e-Millat University (Shifa College of Medicine)/ Shifa International Hospital, Islamabad, Pakistan; 3 Prof. Dr. Munir Iqbal Malik, MBBS, MD. Diplomat American Board of Pediatrics, Professor of Pediatrics, Shifa Tameer-e-Millat University (Shifa College of Medicine)/ Shifa International Hospital, Islamabad, Pakistan

**Keywords:** Choledochal cyst, Clinical presentations in pediatrics age group, Laboratory findings, Surgical outcomes

## Abstract

**Objectives:**

This study was done to compare the clinical features, laboratory findings and surgical outcomes of pediatric patients with choledochal cysts.

**Methods:**

Retrospective review of the hospital records of all pediatric patients admitted with choledochal cysts from 2011 to 2021 were collected and analyzed. Patients were divided into two groups; infant (less than one year age) and pediatric (1 to 16 years) for statistical comparison of two groups.

**Result:**

The study included 34 children, 9 (26.5%) were infant (<1 year) and 25 (73.5%) were more than one year old. Mean age at diagnosis was 15 months with age ranging from 14 days to 16 years. Females were 19 (55.9%) and males were 15 (44.1%). Type-I choledochal cyst was the most common (73.5%), presentation followed by Type-IVA (26.5%) in our patients. Patients from infant group presented with jaundice in 7 (77.7%), and clay-colored stool in 3 (33.3%) as the most common clinical features, while abdominal pain (88%), vomiting (72%), fever (32%) and pancreatitis (32%) were the frequent presentations among older age group. Post-surgical complications of excision of choledochal cyst were observed in 4 (11.7%) patients.

**Conclusion:**

Choledochal cysts have variable presentations depending upon age of the patients. Complete surgical excision of choledochal cyst is the treatment modality of choice and timely surgical management can prevent complications.

## INTRODUCTION

Choledochal cyst (CC) is a rare congenital dilatation of the bile duct that transports bile from the liver to the gall bladder and small intestine.^1^ The incidence of the disease is one in 100,000 to one in 150,000 live birth in western countries. The highest incidence is recorded in Asian population, which is one in 1000. About two-third of these cases are reported from Japan. Incidence in Pakistan is largely unknown with sporadic cases reported.^2^ Although, it can present at any age the majority of case are diagnosed in children.^3^

Choledochal cysts can result in progressive biliary tract obstruction and biliary cirrhosis.^4^ There are different shapes of cysts; cylindrical (fusiform) and spherical (saccular) are the most common. Choledochal cysts can form in the part of the bile duct inside of the liver (intrahepatic) or outside the liver (extra hepatic). There are five basic types of choledochal cysts based on Todani classification method according to anatomical site.^5^ Type-I is cyst of the extra hepatic bile duct, accounting for up to 90% of all choledochal cysts. Type-II is an abnormal pouch or sac opening from the duct. Type-III is a cyst inside the wall of the duodenum. Type-IV consists of two subtypes; IVA cysts of both the intrahepatic and extra hepatic bile ducts and IVB is rare, multiple cysts of extra hepatic duct. Type-V is also called Caroli disease, in which there is combination of intrahepatic cysts with extra hepatic disease.^6^

The exact etiology of CC is largely unknown. However, anomalous pancreatic duct union is reported in up to 70% of cases where the common bile duct (CBD) and pancreatic duct junction occurs outside the duodenum, causing reflux of pancreatic fluid into the biliary tree.^7^ The reflux of pancreatic enzymes into the bile duct from the junction of common bile duct and pancreatic duct results in localized inflammation, weakness and ultimately dilation of bile duct. Approximately 75% of the patient present during pediatric age group. Clinical features of choledochal cyst patients are variable and depend upon the age of patient. The classical triad of abdominal pain, abdominal mass and jaundice is present only in one-third of the patients. This led to delay in diagnosis and severe complications such as malignant transformation, pancreatitis, cholelithiasis and cholangitis.^8^

Studies related to this topic are lacking in our local setup. The aim of the study was to find common presentation and outcome of choledochal cyst in both infantile and older pediatric age group that helps the physician in early diagnosis of disease and prevents late severe complications.

## METHODS

This study was started after getting approval from ethical review board of Shifa International Hospital (IRB # 109-21). Retrospectively, records of all the choledochal cysts pediatric patients admitted in Shifa International Hospital and Shifa Falahee Community Health Center from 2011 to 2021 were collected.

Data was collected on predesigned Performa. Demographic data including name, gender, age at diagnosis and clinical features; fever, nausea, vomiting, abdominal pain, abdominal mass, abdominal distension, jaundice, clay colored stool, dark colored urine, Classic triad and pancreatitis were noted down. In addition, laboratory investigation findings including total and direct bilirubin, alkaline phosphatase (ALP), alanine transaminase (ALT), aspartate transaminase (AST), and prothrombin time (PT), international normalized ratio (INR) serum lipase and amylase. Imaging modalities including ultrasound and CT/MRI abdomen, MRCP (magnetic resonance cholangiopancreatography) were also listed. Patients were divided into two groups; infant (less than one year age) and older pediatric (1 to 16 year age) and all data were compared between these two groups. Patients undergoing surgical procedures were noted.

Statistical analysis was done using Chi-Square test. A p-value of <0.05 was considered significant. Statistical analysis was performed using SPSS 23 for windows.

## RESULTS

The study included 34 children, 9 (26.5%) were diagnosed in infancy and 25 (73.5%) were more than one year old. Mean age at diagnosis was 15 months with minimum age of 14 days of life and maximum age of 16 years. In the infantile group, four patients were neonates with three of them had biliary atresia along with Type-I CC. Regarding gender distribution, females were 19 (55.9%) and males were 15 (44.1%). Consanguinity was reported in three families (8.8%).

Type-I Choledochal cyst was the most commonly (73.5%) reported cyst in our patient population, followed by Type-IVA noted in nine patients (26.5%). Details of patients along with clinical features are shown in Table-I. Most common symptom in infant group was jaundice seen in seven patients (77.7%) followed by clay colored stool in three patients (33.3%). In children age more than one year, abdominal pain and vomiting were predominant complaints as shown in Table-I. The classic triad of abdominal pain, jaundice and palpable abdominal mass was present in only one child. Laboratory workup is shown in Table-II.

**Table-I:** Types and clinical features in two age groups.

	<1 year	>1 year	Number of patients	p-value
***Gender***				0.4
Male	5	10	34	
Female	4	15		
Type of cyst	Type-I 7 Type-IVA 2	Type-I 18 Type-IV 7	34	0.37
Fever	0	8	8	0.05
Vomiting	1	18	19	0.002
Abdominal pain	2	22	24	0.001
Abdominal distension	1	4	5	0.7
Jaundice	7	4	11	0.001
Clay colored stool	3	1	4	0.001
Pancreatitis	1	8	9	0.22
Classic triad	0	1	1	0.55

**Table-II:** Laboratory findings of patients

laboratory	Mean ± SD	No. of patients
Total bilirubin	3.1 ± 3.0	30
Direct bilirubin	2.2 ±2.0	30
INR	1.3 ± 1.0	30
ALT	115 ± 105	32
AST	139 ± 127	31
Alkaline phosphatase (ALP)	473 ± 437	32
Serum amylase	1081 ± 930	11
Serum lipase	1257 ± 1025	15
Prothrombin time	10 ± 6.74	27

Diagnostic imaging modalities used were ultrasound abdomen in 19 (56%) patients, CT abdomen in 13 (38.2%), MRI was done in two (5.9%) patients confirming the diagnosis of CC and identifying the types. All patients underwent surgical correction and post-surgical complications were noted in only four patients, including abdominal pain in two and cholangitis and pancreatitis in one patient each.

## DISCUSSION

The study highlights that infant and older age pediatric patients have different specific symptoms of choledochal cyst. Females were commonly affected in our study population. A study from Karachi showed a similar female (78%) preponderance.^9^ Literature from other parts of the world also support that CC is more commonly seen in females.

Infantile group including neonate specifically presented with obstructive jaundice and acholic stools whereas in older pediatric age group, only five patients had jaundice and acholic stools (p 0.001). Similar results were concluded by other studies in children.^10^ Pediatric patients of older age group specifically presented with abdominal pain, vomiting and fever in comparison to infantile patients (p <0.05). Hung et al and Chen et al. analyzed similar result but according to later vomiting presented equally in both age groups.^10^,^11^ Twenty years study from Taiwan include 25 patients of both infantile and pediatrics age group showed that all eight (100%) patients in the infant group suffered from jaundice and had clay-colored stools whereas only six (35%) patients in the classical pediatric group displayed these features. These findings are consistent with our study population.

Classic triad of abdominal pain, right upper quadrant abdominal mass and vomiting was found in one patient (2.9%) only. Literature supports the fact that it is a rare presentation in children. Similarly, Fumino et al.^12^ found no patient with classical triad and Ohashi et al.^13^ reported the classical triad in 7.47% of their total study population.

In our observation, most of the patients had elevated serum bilirubin level and derange PT/INR especially in infantile group in comparison ALT, AST whereas pediatric age group patients mostly deranged labs were ALT, AST, and serum amylase and lipase levels. Pancreatitis is a serious complication of CC was seen in 9 (26.5%) patients, mainly in older age group.^13^

Choledochal cyst Type-I is the most common type in both age groups followed by Type-IVA. This is in accordance with findings reported from other parts of the world.^14^,^15^ Type-I has been reported in up to 80% of the cases.^16^ Ultrasound abdomen was diagnostic modality in our study in 19 (56%) of the cases, 13 (38%) of cases by CT abdomen and remaining 2 (6%) were diagnosed by MRI/MRCP.^17^ Choledochal cyst can be easily diagnosed by simple imaging modality such as ultrasound abdomen which is most cost effective and convenient. With the use of improved diagnostic modalities like CT and MRI CC are increasingly diagnosed prenatally leading to early diagnosis and improved treatment outcomes.

Cystic excision and Roux-en-Y hepaticojejunostomy were two major surgical procedures done in our patients.^18^ Both of these procedures provide satisfactory results for the vast majority of cases of both age groups and was associated with postoperative complications only in minority of patients.^19^ Early diagnosis and timely surgical correction limits the chances of complications and also shortens the duration to recovery. In this analysis postoperative complications developed only in four patients, two of them having abdominal pain, one each with cholangitis and pancreatitis.^20^

### What is known about the subject?

1. Choledochal cyst (CC) is a rare congenital defect of the bile ducts.
2. It can lead to progressive biliary tract obstruction and biliary cirrhosis.
3. There are five basic types of choledochal cysts based on Todani classification, with Type-I and Type-IVA are frequent.

### What this study adds?

1. The study identifies common presentations and surgical outcomes of choledochal cyst in children that can helps the primary care physicians in early diagnosis.
2. The study highlights that infant and older pediatric age group patients with choledochal cyst have different specific symptoms of presentation.
3. Early diagnosis allows for timely surgical correction and prevent long term complications.

### Limitations:

It includes retrospective nature of the study; limited number of patients and lack of long term follow up findings.

## CONCLUSION

The study aids in different and specific clinical features of choledochal cysts in children. Information gathered through the study is expected to facilitate the physicians in early clinical suspicion of choledochal cyst and prevention of its late complications.

### Author’s Contribution:

**MAF:** Data collection, Manuscript writing and analysis. He is also responsible for the integrity and accuracy of the manuscript.

**SAK:** Manuscript writing and review analysis.

**MIM:** Review analysis.
